# Imaging features of primary sites and metastatic patterns of angiosarcoma

**DOI:** 10.1186/s13244-021-01129-9

**Published:** 2021-12-18

**Authors:** Basrull N. Bhaludin, Khin Thway, Margaret Adejolu, Alexandra Renn, Christian Kelly-Morland, Cyril Fisher, Robin L. Jones, Christina Messiou, Eleanor Moskovic

**Affiliations:** 1 Department of Radiology, The Royal Marsden Hospital, 203 Fulham Rd, London, SW3 6JJ England, UK; 2 Sarcoma Unit, The Royal Marsden Hospital, 203 Fulham Rd, London, SW3 6JJ England, UK; 3 Institute of Cancer Research, London, UK; 4 Department of Musculoskeletal Pathology, Royal Orthopaedic Hospital NHS Foundation Trust, Robert Aitken Institute for Clinical Research, University of Birmingham, B15 2TT Birmingham, UK

**Keywords:** Angiosarcoma, CT, MRI, Metastasis, Radiation

## Abstract

Angiosarcomas are rare, aggressive soft tissue sarcomas originating from endothelial cells of lymphatic or vascular origin and associated with a poor prognosis. The clinical and imaging features of angiosarcomas are heterogeneous with a wide spectrum of findings involving any site of the body, but these most commonly present as cutaneous disease in the head and neck of elderly men. MRI and CT are complementary imaging techniques in assessing the extent of disease, focality and involvement of adjacent anatomical structures at the primary site of disease. CT plays an important role in the evaluation of metastatic disease. Given the wide range of imaging findings, correlation with clinical findings, specific risk factors and patterns of metastatic disease can help narrow the differential diagnosis. The final diagnosis should be confirmed with histopathology and immunohistochemistry in combination with clinical and imaging findings in a multidisciplinary setting with specialist sarcoma expertise. The purpose of this review is to describe the clinical and imaging features of primary sites and metastatic patterns of angiosarcomas utilising CT and MRI.

## Key points

- Angiosarcomas originate from the endothelial cells of lymphatic or vascular origin.
- Imaging presentation is variable and relies on correlation with history and demographics.
- MRI is preferred for the assessment of localised disease pre- and post-treatment.
- CT is utilised for the detection and response assessment of metastatic disease.
- Cystic lung nodules with surrounding ground-glass changes are characteristic of metastatic angiosarcoma.

## Background

Angiosarcomas are rare, aggressive malignant tumours originating from the lymphatic or vascular endothelial cells, making up less than 2% of all soft tissue sarcomas [1, 2]. A large proportion of patients present with advanced or metastatic disease with a reported rate of 16–44%. The median overall survival is poor, ranging from 10 months to 3.4 years [3–7]. They have a heterogeneous clinical presentation and can occur at any age with a median age at diagnosis ranging from 52 to 67 years [3–7]. Men and women are affected equally [1], but some studies have shown that head and neck angiosarcomas occur twice as frequently in men as in women [3–7].

### Aetiology and risk factors

The aetiology is unknown in most cases, but there are several risk factors that are associated with angiosarcomas [8]. Previous radiotherapy is an independent risk factor which has been most commonly described in the breast, but can also occur at any previously irradiated sites for other malignancies [1]. A previous study using data from the Surveillance, Epidemiology and End Results (SEER) registries has shown that radiotherapy is associated with an increased risk in the development of secondary sarcomas in breast cancer patients, with angiosarcomas being the most prevalent accounting for 56.8% of radiation-associated sarcomas [9]. Although the risk is higher following radiotherapy, the incidence of radiation-associated angiosarcomas is still low [10].

Excessive ultraviolet (UV) radiation from prolonged sun exposure has also been implicated as a risk factor for cutaneous angiosarcomas, given that they occur more commonly in the face and scalp of elderly Caucasian men [11, 12].

The relation between angiosarcoma and chronic lymphoedema (also known as Stewart–Treves syndrome) has been historically established in post-mastectomy patients with lymphoedema in the upper limb but in can be of any origin [13]. Any longstanding lymphoedema such as chronic infections or Milroy’s disease is recognised as a risk factor linked to angiosarcoma [1]. Familial syndromes such as Maffucci syndrome, Klippel-Trenaunay syndrome and neurofibromatosis have also been linked to angiosarcoma [1]. A variety of chemicals have also been associated with the development of angiosarcomas, particularly within the liver. Some of these chemicals include the occupational use of vinyl chloride and the use of thorium dioxide for radiological examinations in the past [14]. Angiosarcoma associated with foreign bodies such as metal following orthopaedic procedures [15] and in renal transplant patients either in cutaneous form or at the site of the disused arteriovenous fistula [16] have also been reported.

### Histopathology

**Fig. 1:**
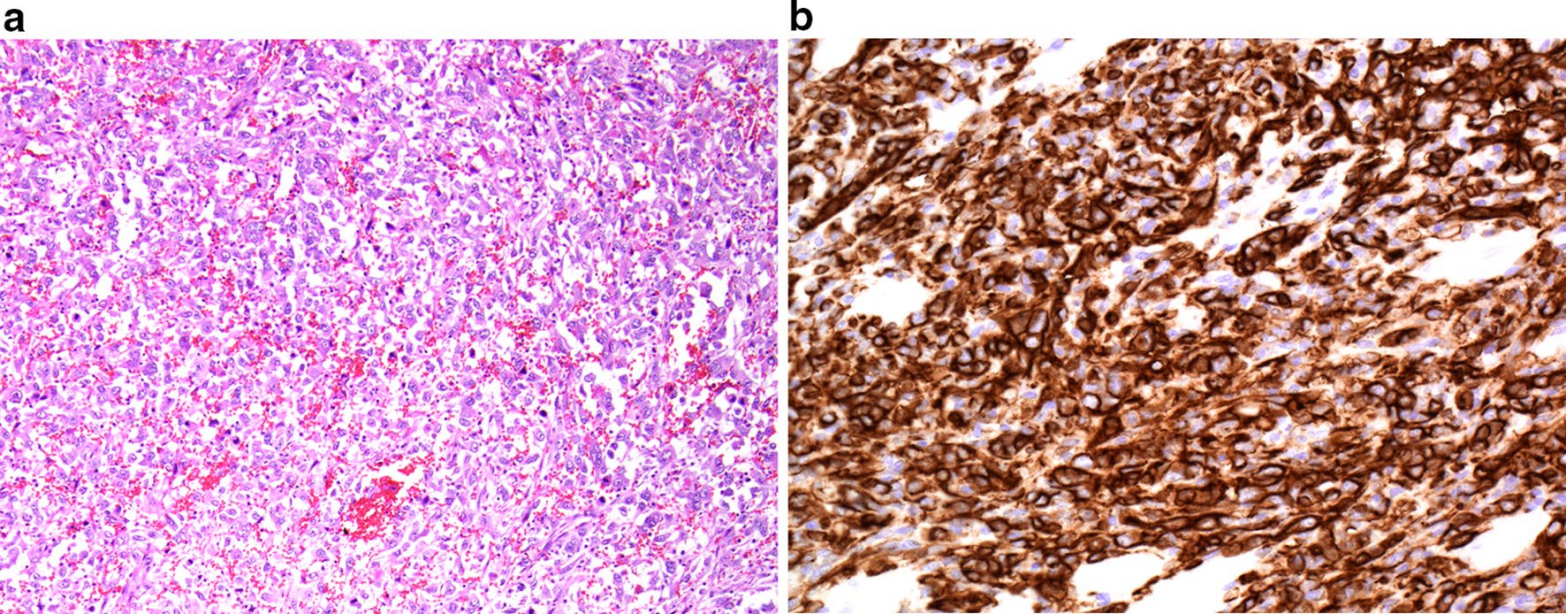
Histopathological features of angiosarcoma. **a** Photomicrograph of histologic specimen shows extensive, poorly formed, anastomosing vascular channels containing prominent blood. The channels are lined by moderately atypical ovoid cells with vesicular nuclei and prominent nucleoli (H and E, × 100). **b** Photomicrograph of histologic specimen shows a predominantly solid architecture without apparent vascular channel formation, but shows diffuse and strong expression of the vascular endothelial marker CD31. CD31 immunohistochemistry is seen to highlight small vascular channels within the mostly solid configuration of the tumour (immunohistochemistry, × 200)

Histologically, features of angiosarcoma are highly variable, and distinguishing benign and malignant vascular tumours can be challenging on light microscopy. The spectrum of findings can range from malignant endothelial cells forming vascular sinusoids which can dissect through collagen bundles, to solid sheets of spindle or epithelioid cells without definite vasoformation [1, 2, 8]. When epithelioid endothelial cells predominate, these tumours are classified as epithelioid angiosarcomas, which are poorly differentiated with areas of haemorrhage and necrosis and associated with a poorer prognosis [2, 17]. Given the challenges in the histological diagnosis, immunohistochemistry is important in confirming the diagnosis. According to the 2020 WHO classification of soft tissue tumours, the essential diagnostic criteria include the expression of CD31 and ERG on immunohistochemistry [8]. Other histopathological features specified in the essential criteria are vasoformative or sheet-like growth, multilayering of endothelial cells, nuclear atypia, increased mitoses and necrosis (Fig. 1) [8].

According to Young et al. [1], angiosarcomas can be classified into several groups: cutaneous, lymphoedema-associated, radiation-associated, primary breast and soft tissue angiosarcomas. They can arise from any part of the body, but the commonest location is the head and neck, followed by the breast. Some studies have indicated that the tumour behaviour is dependent on the primary site, with a poorer prognosis associated with deep/visceral location of the site of origin [4, 6].

**Table 1:** Imaging findings of primary hepatic angiosarcoma

	Yi [50]	Pickhardt [49]	Bruegel [48]	Koyama [51]	White [14]
No. of patients	19	35	7	13	5
Focality	Predominantly unifocal	All multifocal	Predominantly multifocal	Predominantly multifocal	Predominantly multifocal
Tumour border	Indistinct	Well-circumscribed	N/A	N/A	N/A
Size	3–18 cm	2.6–20 cm	< 3–19 cm	< 3–14 cm	N/A
*CT*					
Non-enhanced	Homogeneous/heterogeneous low density with focal areas of hyper attenuation	Heterogeneous density with focal areas of hyper attenuation	Hypodense with focal areas of hyper attenuation	Heterogeneous low density with focal areas of hyper attenuation	Hypodense
*MRI*					
T1	Heterogeneously low SI	N/A	Low SI with focal areas of high SI	Low SI with focal areas of high SI	N/A
T2	Heterogeneously high SI	Predominantly high SI	Heterogeneously high SI	Heterogeneously high SI	N/A
Contrast-enhancement pattern	Heterogeneous enhancement with centripetal filling	Heterogeneous foci of hypervascular enhancement with variable morphology but with progressive expansion on portal venous phase and follow blood pool on more delayed phase	Variable patterns at early phase, including patchy peripheral rim enhancement, bizarre-shaped intralesional foci of enhancement and small lesions with no enhancement. On dynamic contrast imaging, lesions showed varying degrees of progressive enhancement, with small nodules showing homogeneous enhancement	Variable patterns of enhancement depending on morphology – multiple small nodules showed hypoattenuating nodules; large masses showed heterogeneous enhancement with central necrosis. Progressive enhancement over time on delayed post-contrast imaging except for central regions	Intense and progressive enhancement in vascular areas of tumour, either peripheral or central
Cirrhosis	4	15	N/A	N/A	N/A
Metastasis at presentation	3	16	2	7	N/A

The aim of this review is to present the clinical and imaging findings of angiosarcoma of different anatomical sites and to demonstrate the metastatic patterns of angiosarcoma utilising CT and MRI. The cases presented in this review are drawn from 399 cases of histologically confirmed angiosarcomas referred to the Sarcoma Unit at The Royal Marsden Hospital over a 10-year period from 1 January 2011 to 31 December 2020.

## Role of imaging

MRI is the preferred modality of choice for the assessment of localised soft tissue sarcomas [18]. It provides detailed soft tissue contrast with high spatial resolution. It can also demonstrate anatomical information, disease extent and tumour composition. Advanced MRI sequences such as diffusion-weighted imaging (DWI) allow assessment of tumour cellularity which helps delineate extent as well as treatment response assessment [19]. At our institution, the recommended protocol includes T1- and T2-weighted imaging, short tau inversion recovery, T1-weighted fat-saturated imaging pre- and post-intravenous contrast and DWI with apparent-diffusion coefficient (ADC) maps. In the breast and liver, dynamic contrast-enhanced (DCE) MRI sequences are included to allow examination of the contrast enhancement pattern which will be described later in this article. There are some disadvantages in using MRI, however, which include longer scanning times, limited scanner capacity, and higher costs compared to CT.

CT with intravenous contrast is most useful for staging and response assessment of metastatic disease. It is easily accessible and cheaper than MRI or PET-CT. CT can be useful for assessing for bony involvement and detecting the presence of calcifications. The disadvantages of using CT include exposure to ionising radiation and poorer soft tissue contrast compared to MRI.

In the breast, mammography and ultrasound are not particularly helpful due to their non-specific findings, and the diagnosis should be confirmed with a punch or percutaneous biopsy, depending on whether the lesion is superficial or deep. Ultrasound can be useful in some cases, for example in the initial assessment of suspected aggressive deeper masses in the extremities. In this setting, ultrasound can also provide information on tumour vascularity and suitability of a percutaneous biopsy.

In the UK, the use of positron emission tomography-computed tomography (PET-CT) is currently not part of routine investigation in soft tissue sarcoma although it may be considered in some cases such as prior to radical surgery [20].

## Areas of involvement

### Head and neck

The head and neck is the commonest primary anatomic site, accounting for 27% of all angiosarcomas [1]. Head and neck angiosarcomas most commonly occur in the elderly with a peak presentation in the seventh decade and are more common in men [1, 3, 7, 21]. Previous data have indicated an association between excessive UV light exposure and cutaneous angiosarcomas as they are frequently seen in the sun-exposed face and scalp of elderly Caucasian men [11, 12]. Fifty percent of cutaneous angiosarcomas occur in the head and neck, mainly in the scalp and cheek [12]. The early presentation of cutaneous angiosarcoma can resemble a bruise and is easily misdiagnosed as a benign lesion, leading to a delayed diagnosis. At later stages, the tumour can develop into a fungating, ulcerating and bleeding lesion [1].

**Fig. 2:**
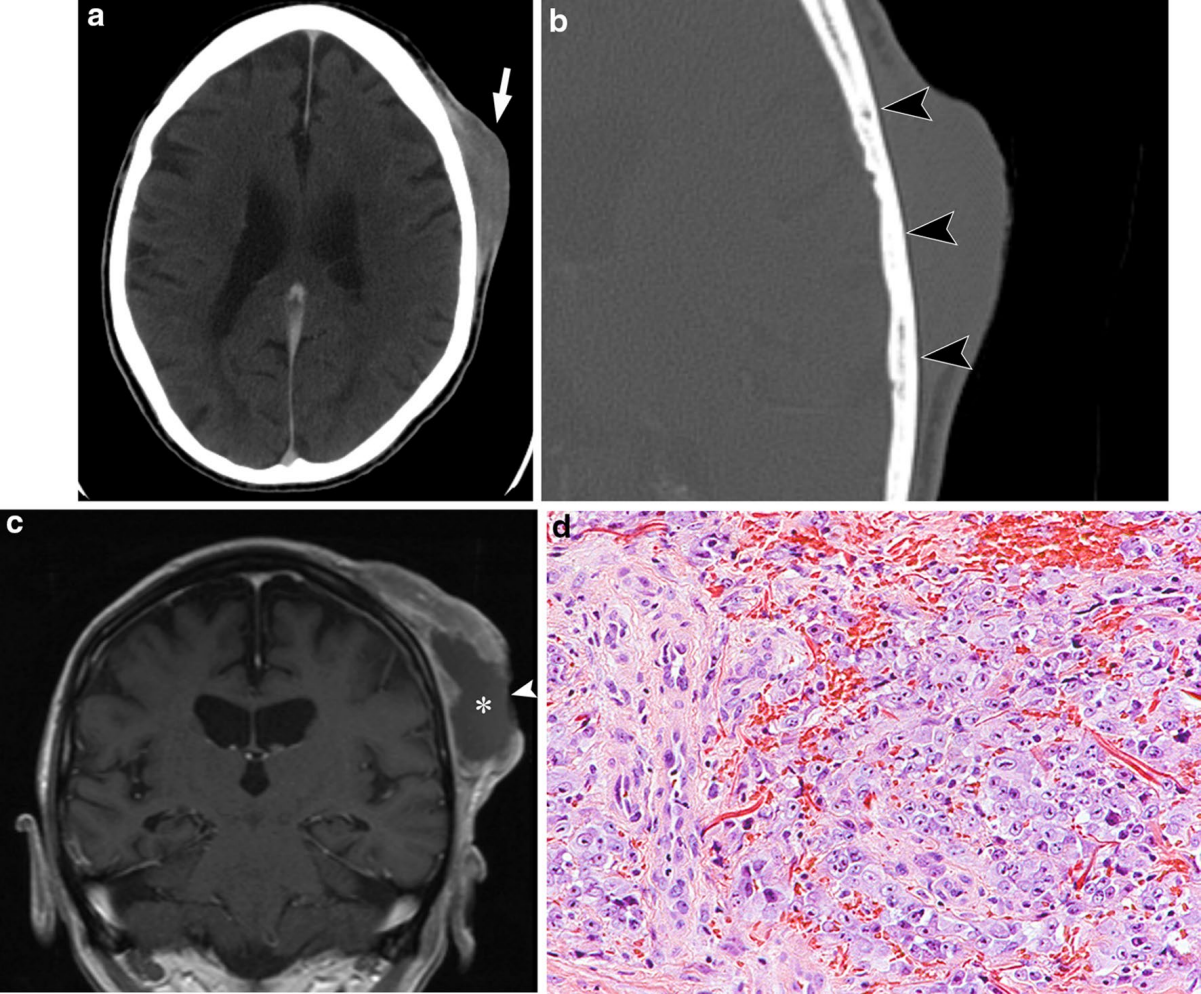
77-year-old man with cutaneous scalp angiosarcoma. **a** Axial contrast-enhanced CT images show a lobulated heterogeneous cutaneous mass overlying the left scalp (arrow) with infiltration of the subcutaneous tissues and contacting the outer table of the skull. **b** Axial CT (bone windows) image shows no evidence of bony destruction (arrowheads). **c** Coronal contrast-enhanced T1-weight MR image performed 3 months later shows enlargement of the cutaneous mass. There is heterogeneous peripheral enhancement of the mass lesion with central necrosis (asterisk) and ulceration (arrowhead). **d** Photomicrograph shows a specimen mostly composed of large, anaplastic epithelioid cells with extensive surrounding haemorrhage. Only focally (left of field), vasoformation is evident, with variably sized, sometimes compressed vessels lined by similarly markedly atypical ovoid cells with prominent nuclei (H and E, × 200)

Cutaneous thickening and infiltration of the subcutaneous fat can be seen on CT [22, 23]. CT can be used to assess for bony involvement [22] particularly if radical surgery is being considered. On MRI, angiosarcoma lesions show intermediate T1 and high T2 signal intensity with aggressive infiltration of the adjacent tissues and avid heterogeneous contrast enhancement (Fig. 2) [21]. Other features may also include haemorrhage, necrosis and flow voids with low signal intensity in all pulse sequences due to high flow [24].

Differentiating between benign and malignant lesions in the head and neck can be very challenging [21]. The imaging features for angiosarcoma are non-specific, and correlation with clinical history, risk factors and demographics is crucial. The presence of a painful enlarging cutaneous mass in the head and neck in an elderly man is an important clue to the diagnosis, although another differential diagnosis to consider is Kaposi sarcoma if there is a history of acquired immunodeficiency syndrome (AIDS).

### Extremities

Pooled data from 534 patients from several studies show that 15.3% of all angiosarcomas occur in the extremities [1]. Angiosarcomas of the extremities can develop in the setting of chronic lymphoedema (Stewart–Treves syndrome), sporadically or after previous radiotherapy [25]. The tumours occur in the skin and subcutaneous tissues with the appearance of multifocal ill-defined spreading bruises [25]. The lesions can also develop in the deep compartments of the extremities, and isolated case reports of deep soft tissue angiosarcoma within the extremities are of the epithelioid subtype [26, 27]. The pathogenesis of angiosarcoma in the setting of chronic lymphoedema is unclear. It has been proposed that the oedematous region behaves as an “immunologically privileged site”, whereby tumour development is able to take place at this site without triggering a sufficient immune response [28].

**Fig. 3:**
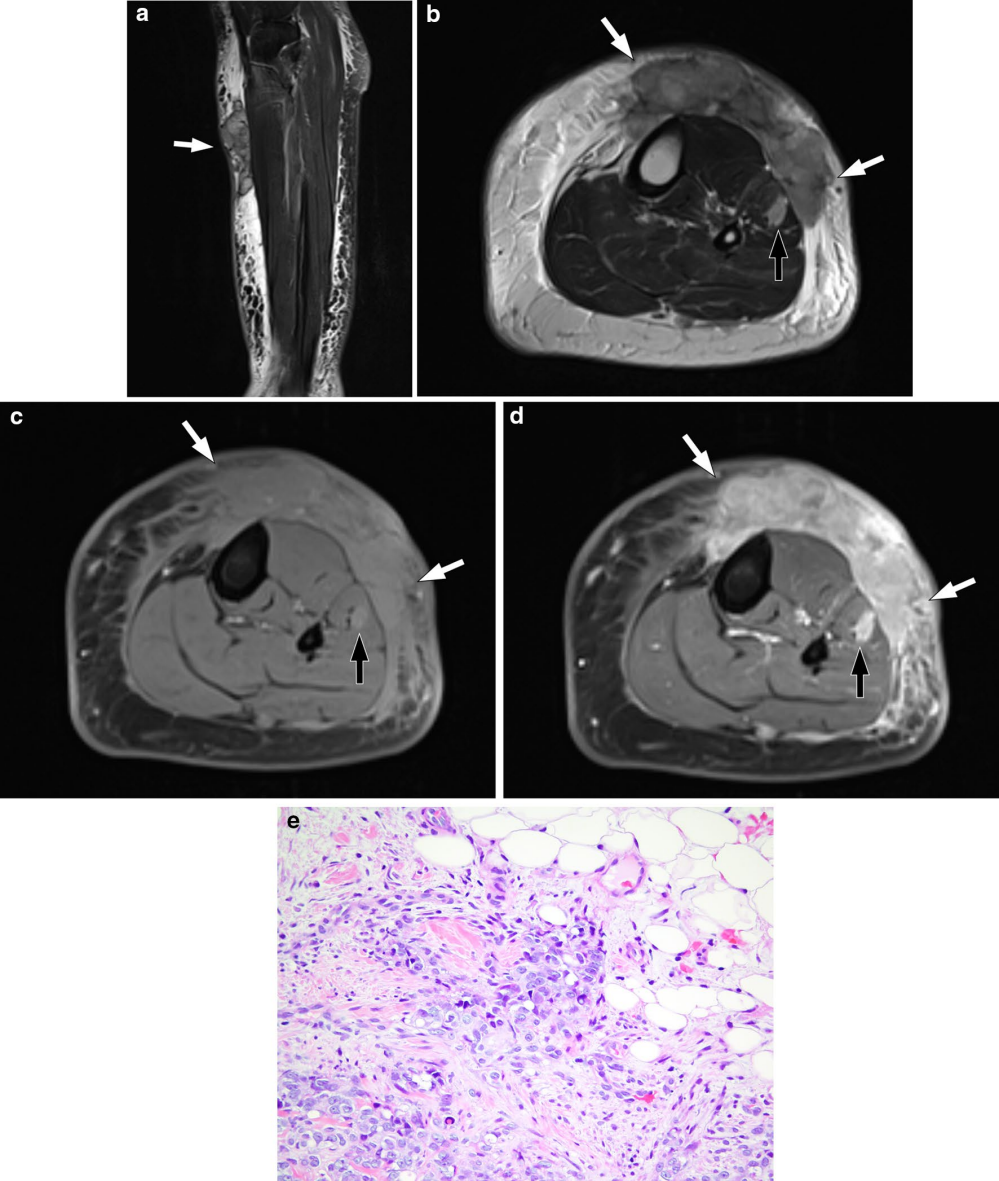
53-year-old woman with angiosarcoma associated with congenital lymphoedema of the left leg (Stewart–Treves syndrome). **a** Sagittal T2-weighted fat-suppressed image shows diffuse circumferential oedematous changes within the subcutaneous tissues and skin thickening in the left leg. There are multiple well-circumscribed nodules in the anterior proximal leg which are of predominantly low signal intensity compared to the surrounding tissues (arrow). **b**–**d** Axial images of the left leg show skin thickening and multiple soft tissue nodules (white arrows) within the oedematous subcutaneous tissues with a further intramuscular nodule (black arrow) within the lateral muscular compartment of the leg. **b** Axial T2-weighted image shows the nodules to be of heterogeneously lower signal compared to the adjacent oedematous subcutaneous tissues and higher signal compared to the skeletal muscles with central low signal striations. **c** Axial T1-weighted fat-suppressed pre-contrast image shows the nodules to be isointense compared to the skeletal muscles. **d** Axial T1-weighted fat-suppressed post-contrast image shows avid heterogeneous enhancement of the nodules within the subcutaneous tissues (white arrows) and the lateral muscular compartment (black arrow). **e** Photomicrograph shows a specimen composed predominantly of epithelioid cells, is seen to prominently infiltrate the dermal collagen and the subcutaneous adipose tissue (top right). Much of the tumour is disposed in solid nests, but small areas of vasoformation are discernible (H and E, × 200)

On CT, multiple cutaneous nodules with increased density of the subcutaneous adipose tissues and exuberant skin thickening have been reported of lymphoedema-associated angiosarcoma [29]. On MRI, the nodules are isointense to skeletal muscle on T1 and are of predominantly low signal intensity on T2 with avid contrast enhancement on a background of circumferential skin thickening [30–32]. The low signal intensity on T2 and avid contrast enhancement of the lesion allows the tumour to be more readily visible particularly in the setting of chronic lymphoedema with diffuse background high T2 signal within the oedematous tissues (Fig. 3) [32]. Low T2 signal intensity is an unusual imaging feature for a highly vascular tumour and is postulated to reflect the prominence of fibrous stroma with densely packed tumour cells and paucity of small vessels on histology [30, 31].

Given that a majority of angiosarcomas in the extremities are superficial in nature, the diagnosis is made clinically and confirmed with a punch biopsy before imaging is undertaken. Imaging may be performed following histological confirmation if assessment of local disease extent is required for treatment planning, or for surveillance in a post-treatment setting.

### Breast

The breast is one of the most common sites for angiosarcomas to develop and can be categorised into primary and secondary angiosarcomas [33].

**Fig. 4:**
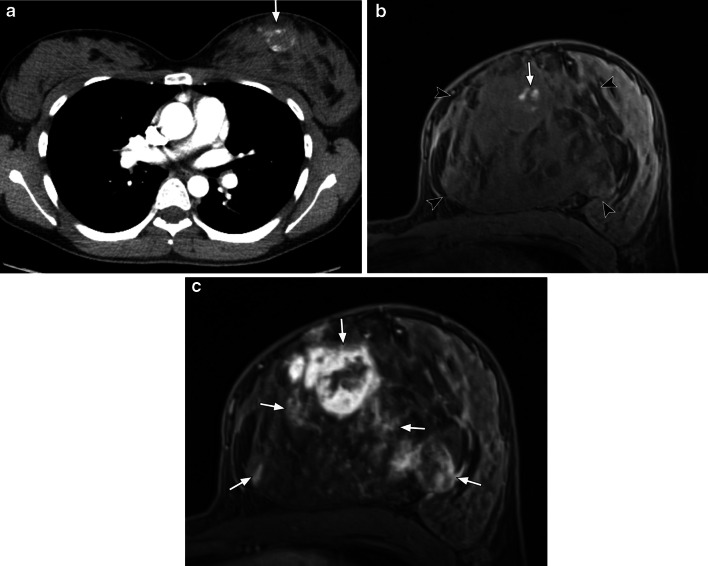
27-year-old woman with primary left breast angiosarcoma. **a** Contrast-enhanced axial CT image shows a lesion in the medial left breast with peripheral rim enhancement (arrow). **b** Axial T1-weighted fat-suppressed pre-contrast image shows a large mass (arrowheads) occupying much of the left medial and central left breast which displaces the normal fibroglandular tissues laterally. There is a small area of high T1 signal within the mass suggestive of haemorrhage (arrow). **c** Axial T1-weighted fat-suppressed early dynamic post-contrast image shows multifocal areas of variable heterogeneous peripheral enhancement (arrows). The extent of disease was underestimated on CT

Primary breast angiosarcomas account for 0.04% of all breast tumours and 8% of breast sarcomas [34]. They are more frequently seen in younger women in the third–fourth decades of life and typically arise within the breast parenchyma [33]. A common presentation is a rapidly growing palpable mass, with up to a third associated with bluish skin discolouration. On mammography, the most common finding is an ill-defined, non-calcified mass or focal asymmetry, with a mean size of 5.9 cm at presentation [34]. Approximately 33% of angiosarcomas are not detected on mammography due to the younger age group which have denser background parenchyma [35]. MRI typically shows a heterogeneous lobular mass with low signal intensity on T1, high signal intensity on T2 and brisk heterogeneous enhancement with washout kinetics (Fig. 4) [34]. High T1 signal intensity areas may also be evident, representing haemorrhage or venous lakes [34].

**Fig. 5:**
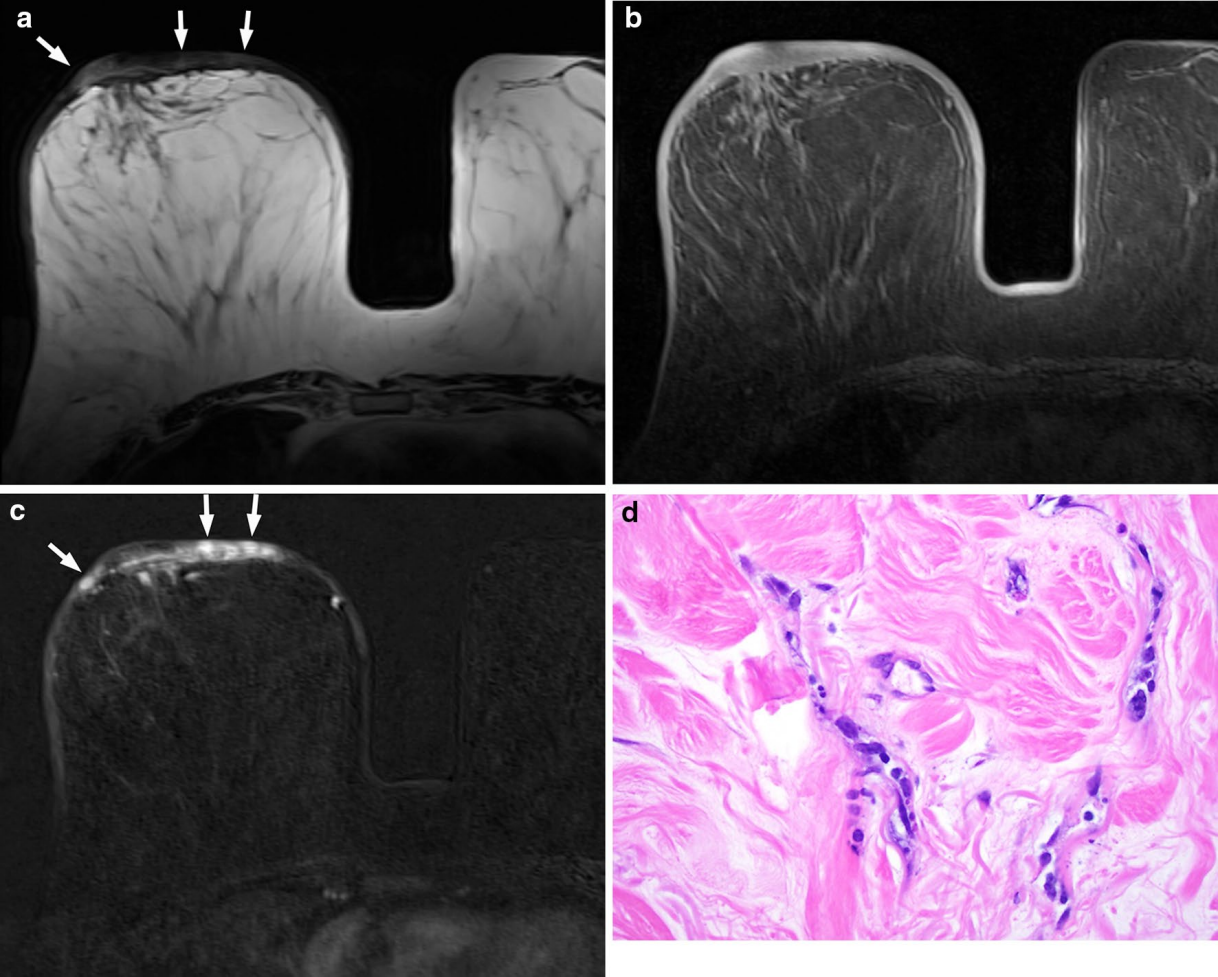
62-year-old female with radiation-associated angiosarcoma of the right breast. She had a previous grade 1 invasive ductal carcinoma of the right breast 10 years earlier which was treated with breast-conserving surgery and radiotherapy. **a** Axial T2-weighted image shows unilateral cutaneous thickening with areas of high signal intensity (arrows) in the right breast. **b** Axial T1-weighted fat-suppressed pre-contrast image shows isointense thickening of the right breast compared to the left. **c** Axial T1-weighted fat-suppressed early post-contrast subtracted image shows multifocal areas of avid enhancement within the thickened cutaneous layer. **d** Photomicrograph of the skin of the breast shows small, compressed slightly angulated vessels with minimally atypical hyperchromatic ovoid to spindle nuclei, with nuclear debris. These vessels are seen to dissect the surrounding dermal collage (H and E, × 400)

Secondary or radiation-associated angiosarcomas (RAS) of the breast are more frequently seen in older women who have undergone treatment for breast cancer, with a median age of 70 [36]. Incidence of RAS ranges from 0.14% to 0.5% [37, 38] with an average time of 6 years (range between 1 and 41 years) between radiotherapy and the development of angiosarcoma [33, 36]. RAS often develop in the cutaneous and subcutaneous tissues of irradiated skin of the breast or chest wall within the radiotherapy field, but can also occur within the breast parenchyma [39]. Due to the predominant skin involvement, the presentation is similar to that of cutaneous angiosarcoma with red plaques or skin discolouration which could be mistaken for bruises. Findings on mammography can be skin thickening only which is non-specific and difficult to differentiate from expected post-radiotherapy changes [36]. Confirmation with a punch biopsy is therefore crucial if there is any clinical suspicion. A retrospective study by Chikarmane et al. has shown that there was skin thickening associated with high T2 signal intensity in all RAS patients on MRI, although nearly half demonstrate discrete hypointense lesions [40]. One quarter had intra-parenchymal masses, and all lesions showed rapid contrast enhancement with washout kinetics (Fig. 5) [40].

### Cardiac

Primary cardiac malignancies are rare with cardiac metastases being 20–40 times more common, but angiosarcoma is the most common primary malignancy of the heart and pericardium [41]. Cardiac angiosarcomas can occur at any age but more frequently found in patients between the age of 30–50 years, with a male/female ratio of 2:1 [42]. The clinical signs and symptoms are non-specific, but these include chest pain and shortness of breath related to pericardial effusion, dyspnoea and syncope. 80% of cardiac angiosarcomas arise in the right atrium [41] and frequently extend to the pericardium, vena cava or tricuspid valve [43]. The prognosis is poor given the unfavourable anatomic location of the tumours, aggressive biological behaviour and high propensity to metastasise. The mean overall survival time is approximately 4 months, but with surgical excision, can improve to 10 months [43].

**Fig. 6:**
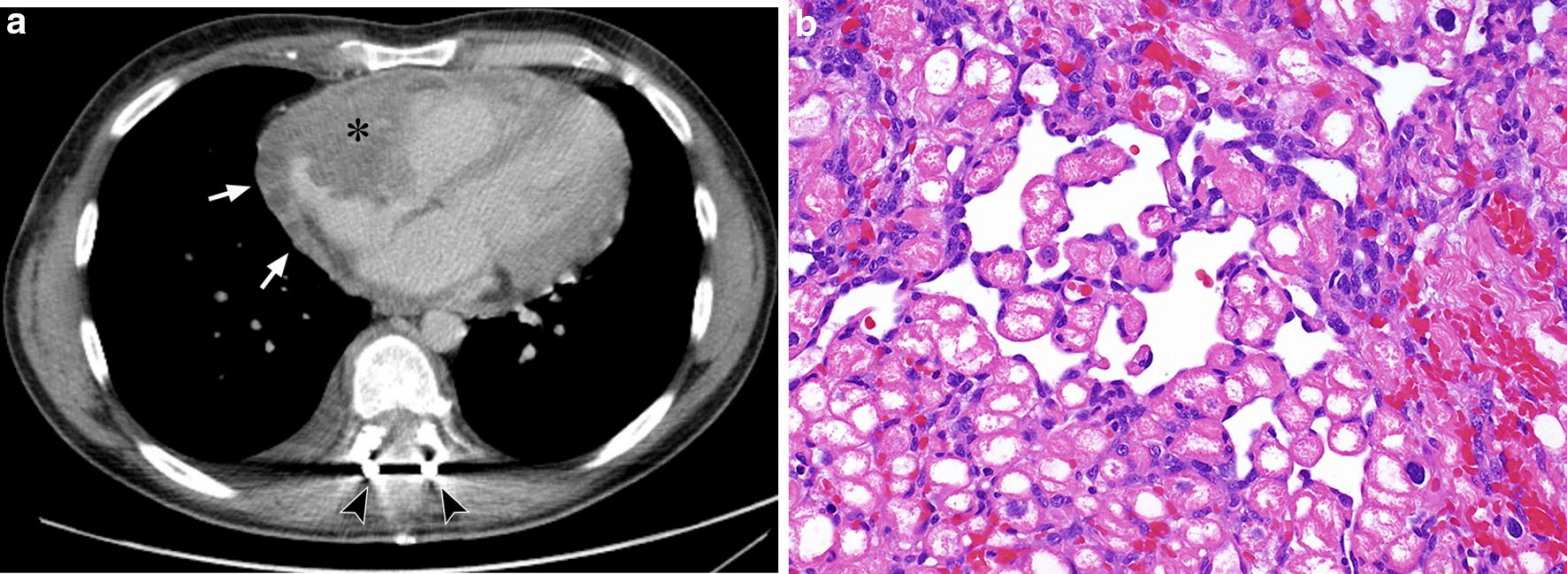
35-year-old man with cardiac angiosarcoma. **a** Axial contrast-enhanced CT shows an irregular mass arising from the right atrial wall protruding into the cardiac chamber (asterisk) with nodular soft tissue extending into the adjacent pericardium (arrows). The patient previously presented with cord compression from a thoracic vertebral metastasis which has been decompressed surgically, as shown by the presence of the spinal fixation rods (arrowheads). **b** Photomicrograph of histologic specimen shows markedly infiltrative tumour extensively permeating cardiac tissue, with dissection of the cardiac myocytes. The minimally atypical spindle and ovoid malignant endothelial cells are seen to line ill-defined, angulated vascular channels insinuating between individual cardiac muscle cells (H and E, × 400)

Two common patterns of growth have been described in the literature. The more common pattern is a large, well-defined mural mass replacing the atrial wall which protrudes into the cardiac chamber [43]. On CT, a filling defect is typically seen in the right atrium representing the mass which may be irregular or nodular in outline (Fig. 6). On MRI, haemorrhagic or necrotic areas may be visible with high signal intensity within the tumour representing blood products [44]. Other case reports have also documented nodular ‘cauliflower’ appearances of the primary tumour or ‘sunray’ appearance of pericardial involvement on MRI [44, 45]. The less common pattern is extensive pericardial involvement without the presence of an atrial component [43]. On CT and MRI, pericardial effusion or thickening may be seen.

Given the non-specific appearances on imaging, there is a wide range of differential diagnosis which may demonstrate similar appearances such as metastases, thrombus, lymphoma, myocarditis and rhabdomyosarcoma [41, 46]. It is noted that the more common atrial myxomas typically arise in the left atrium, but left-sided angiosarcomas have been reported in a very small number of cases and can potentially mimic atrial myxomas [47].

### Liver

Although primary hepatic angiosarcoma only accounts for 2% of primary hepatic tumours, it is the most common mesenchymal malignancy in the liver [48]. It is often associated with a poor prognosis with a median survival of 6 months without treatment [48]. Early reports of hepatic angiosarcoma have demonstrated an association with exposure to chemical agents such as thorium dioxide and vinyl chloride [14], although more recent studies show fewer cases associated with such exposure [49, 50]. Due to the aggressive biological behaviour of this tumour, up to 54% patients have metastatic disease at presentation [14, 48, 51]. Common clinical symptoms include upper quadrant pain, abdominal discomfort, anorexia and weight loss [50, 51].

Hepatic angiosarcomas can present in several different growth patterns—single dominant large mass, multiple nodules, a combination of a dominant mass with nodules, and diffuse infiltrative nodules [50, 52]. A majority of the published retrospective cases series show multifocality at presentation [14, 48, 49, 51] with one more recent study showing predominance of the unifocal morphology in the Chinese population [50].

**Fig. 7:**
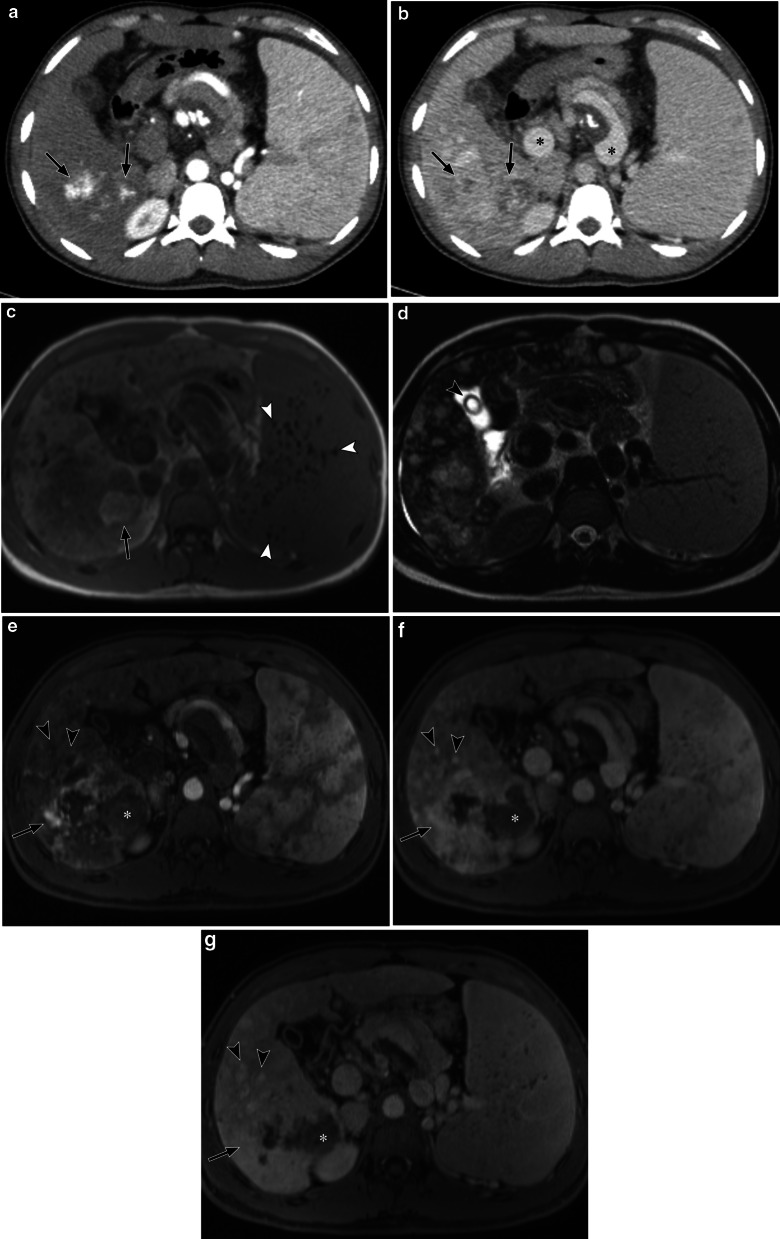
30-year-old man with primary hepatic angiosarcoma and longstanding portal hypertension from microvascular veno-occlusive disease. **a**, **b** Axial contrast-enhanced CT images of the abdomen show a dominant mass in the posterior right hepatic lobe with smaller multifocal nodules. **a** Arterial phase CT image shows central foci of avid enhancement within the dominant lesion (arrows). **b** Portal venous phase CT shows progressive heterogeneous enhancement (arrows). There are features of portal hypertension with distension of the portal and hepatic veins (asterisks) and splenomegaly. **c** Axial T1-weighted in-phase image shows multifocal predominantly low signal intensity lesions compared to the adjacent liver parenchyma. There is a focus of high signal intensity within the large lesion posteriorly suggestive of haemorrhage (arrow). Small low T1 signal nodules within the enlarged spleen represent haemosiderin deposition related to portal hypertension (arrowheads). **d** Axial T2-weighted image shows multifocal heterogeneous hepatic lesions which are of predominantly high signal intensity. There is an incidental calculus within the gallbladder (arrowhead). **e**–**g** Axial T1-weighted dynamic contrast-enhanced MR images of the liver. Arterial (**e**), portal venous (**f**) and delayed-phase (**g**) images show peripheral nodular enhancement of the dominant lesion with progressive enhancement (arrow) and a persistent area of non-enhancement (asterisk). Smaller nodules elsewhere in the liver also show progressive enhancement but appear relatively homogeneous compared to the dominant lesion (arrowheads)

There is a variable pattern demonstrated on CT and MRI, as summarised in Table 1. The variability in the imaging characteristics reflects the heterogeneous cellular configuration of angiosarcoma on histology [51]. One common feature in most of these studies is the presence of hypodensity within the mass with focal areas of hyperattenuation suggesting the presence of haemorrhage on unenhanced CT [48–51, 53]. On dynamic post-contrast imaging with CT and MRI, the pattern of enhancement is variable depending on the morphology of the masses/nodules, but a vast majority of angiosarcomas demonstrated heterogeneous progressive enhancement on the delayed phase (Fig. 7). The lack of rapid contrast wash-out is a useful characteristic feature that could help distinguish angiosarcoma from other entities such as hepatocellular carcinoma or hypervascular metastasis [49, 50, 54].

Progressive enhancement of angiosarcomas, however, can be mistaken for other lesions such as epithelioid haemangioendotheliomas (EHE) and haemangiomas. A retrospective study by Kim et. al. [55] comparing the MRI characteristics of hepatic angiosarcoma and haemangioma demonstrated several distinguishing imaging features. Angiosarcomas are more likely to show multifocal lesions, prominent vessels within the tumour, vascular invasion, enhancing foci of unusual shapes and a range of different enhancement patterns with none reaching complete lesional enhancement [55]. Haemangiomas, on the other hand, are less likely to be multifocal with a vast majority of the lesions showing predominantly centripetal enhancement with complete fill-in centrally on the delayed post-contrast phases [55].

### Spleen

Primary splenic angiosarcoma is exceptionally rare, with an annual incidence of 0.14–0.25 cases per million [56, 57]. There is a slight predominance in men and a median age of 59–63 at presentation [56, 57]. Common symptoms at presentation include splenomegaly, abdominal pain and non-specific systemic symptoms such as fever, fatigue and weight loss [56]. Spontaneous rupture of the spleen is also a common complication, seen in up to 30% of patients [58]. Primary splenic angiosarcoma is associated with a poor prognosis with a majority of patients dying from the disease within 12 months [56, 57].

On CT, there are a number of features which may indicate the diagnosis of splenic angiosarcoma. The commonest findings are splenomegaly with a spleen size measuring over 12 cm and diffuse infiltration by a large heterogeneous complex mass (or masses), but multiple discrete masses, a solitary mass or a poorly defined mass associated with intraperitoneal haemorrhage may also be seen [59]. On non-contrast CT, the lesion may show areas of high density corresponding to areas of acute haemorrhage [58, 59]. On contrast-enhanced CT, the lesion shows peripheral contrast enhancement, similar to the pattern of hepatic haemangioma [59, 60].

On MRI, angiosarcomas can show nodular hypointense signal on T1 and T2-weighted images. Large masses can also show high T1 and T2 signal intensity representing subacute haemorrhage or necrosis. Areas of low T1 and T2 signal intensity may also be seen if there is haemosiderin deposition from chronic haemorrhage [58, 59]. On contrast-enhanced MRI, there is heterogeneous peripheral rim enhancement with no enhancement centrally [59].

**Fig. 8:**
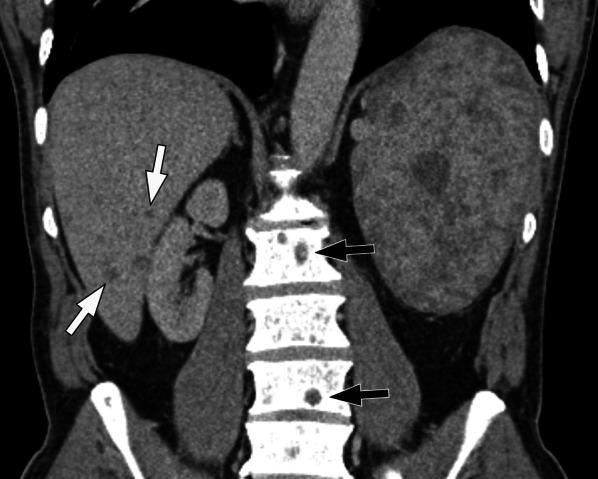
66-year-old man with primary splenic angiosarcoma. Coronal contrast-enhanced CT shows multiple low attenuation lesions of varying sizes within an enlarged spleen. There are multiple low attenuation nodules within the liver (white arrows) and lytic lesions (black arrows) within the vertebral bodies in keeping with liver and skeletal metastases

A retrospective study by Thompson et al. indicates that a diagnosis of a primary malignant splenic tumour can be diagnosed in 10 of 12 patients (83%) in their series [59]. The imaging findings which supported this observation were the presence of splenomegaly with a large mass or multiple splenic lesions, and a majority the cases presented with metastatic disease in the liver or spine (Fig. 8) [59]. Therefore, a combination of these findings would place primary angiosarcoma high on the differential diagnosis list. Other potential differential diagnosis to consider would include other vascular splenic tumours such as haemangioma, littoral cell tumour, lymphangioma, haemangiopericytoma and epithelioid vascular tumours [59]. In our experience, it is often difficult to determine the primary site of disease when there is multifocal disease seen in both the liver and spleen especially given the high rate of metastatic disease at presentation.

### Bones

Angiosarcomas of the bone are very rare, accounting for less than 1% of primary malignant bone tumours and approximately 6% of angiosarcomas occur in the bones [61]. The peak age at presentation is in the third to fifth decades of life, and men are twice as frequently affected than women [61]. Frequent presenting symptoms are pain and swelling at the affected site. The location of bone angiosarcoma is more commonly in the long bones (60%), particularly the tibia (23%), femur (18%), humerus (13%) and pelvis (7%) [62].

The histological and biological behaviour of malignant vascular bone lesions is variable with a myriad of names used to describe each entity, which consists of haemangioendothelioma, angiosarcoma and epithelioid haemangioendothelioma [63]. It has been proposed that the term angiosarcoma should be used to describe the high-grade or poorly differentiated end of the spectrum of malignant vascular bone lesions [63]. The radiological appearances of angiosarcoma and haemangioendothelioma are also non-specific with no distinguishing features [62], which reflects the pathological overlap of these tumours and makes the diagnosis challenging.

**Fig. 9:**
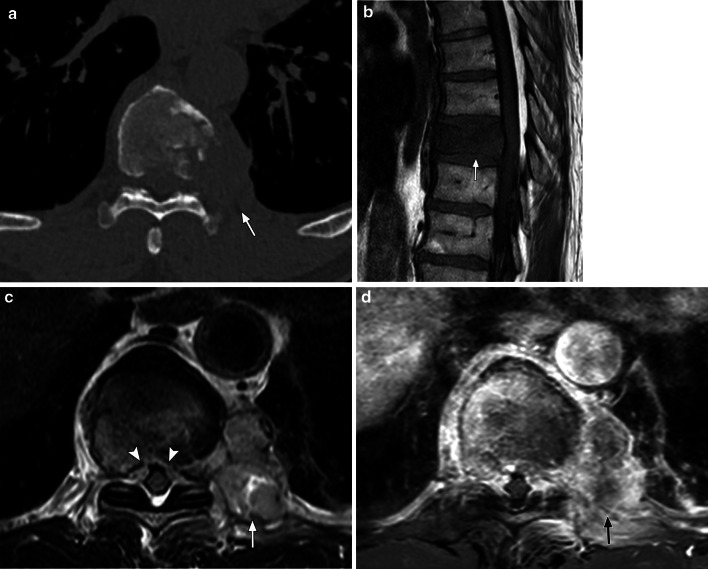
61-year-old man with primary bone angiosarcoma. **a** Axial CT (bone windows) image shows a destructive lesion centred at the left T9 vertebral body with extra-osseous soft tissue (arrow). **b** Sagittal T1-weighted image shows low signal change of the T9 vertebral body (arrow) and bulging of the posterior cortex in keeping with malignant infiltration. **c** Axial T2-weighted image shows infiltration of the T9 vertebral body with a heterogeneous, predominantly high signal intensity left paravertebral mass (arrow) involving the adjacent rib and pleura. There is extra-osseous soft tissue narrowing the spinal canal (arrowheads). **d** Axial T1 fat-suppressed post-contrast image shows avid heterogeneous enhancement of the T9 vertebral body and the left paravertebral mass (arrow)

On plain radiography and CT, the appearances of malignant vascular bone tumours are variable, but the lesions are usually lytic with ill-defined margins (Fig. 9) [63]. Other common findings include endosteal erosion, cortical destruction and extra-osseous tumour extension with no periosteal reaction [63]. A retrospective study of 63 patients with malignant vascular bone lesions showed multifocal lesions in 40% of cases, but the presence of multifocal lesions without periosteal reaction can also be seen in lytic bony metastases or multiple myeloma especially in a patient over 40 years old and should be considered in the differential diagnosis [64]. It has been suggested that if a patient over the age of 40 presents with a well-defined osteolytic lesion over 4 cm in the femur with geographic cortical destruction and no evidence of periosteal reaction, a malignant vascular bone lesion should be suspected [64].

On MRI, malignant vascular bone tumours show low to intermediate T1 signal intensity and high T2 and STIR signal intensities, with heterogeneous contrast enhancement (Fig. 9) [64]. Areas of high T1 signal intensity and fluid–fluid levels suggestive of haemorrhage may also be visible [62]. Extensive ill-defined high T2 signal surrounding the bone and soft tissues was observed in 58% of malignant vascular bony lesions in the study by Vermaat et. al., suggesting intense reactive changes and this feature is not usually seen in metastatic bony lesions [64]. The presence of such intense reactive change has been suggested to be a potential distinguishing imaging characteristic from metastatic bony deposits although this has not yet been confirmed histologically.

### Uncommon sites

Angiosarcomas can occur in the kidney, although these are very rare with less than 40 cases documented in the literature [65]. The peak incidence is in the seventh decade and it is more common in men [65]. The most common presenting symptoms are flank pain and haematuria, although low-grade fever and weight loss have also been reported [66].

**Fig. 10:**
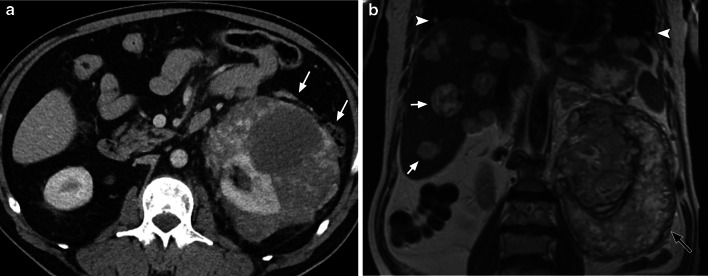
74-year-old man with angiosarcoma of the left kidney. **a** Axial contrast-enhanced CT image shows a large heterogeneously enhancing mass surrounding the left kidney occupying much of the peri-renal space displacing the adjacent mesenteric fat and bowel loops (arrows). There are numerous areas of low attenuation within the mass in keeping with necrosis. **b** Coronal T2-weighted image shows a large mass within the left peri-renal space (black arrow) consisting of predominantly high signal intensity, with low signal intensity striations within the mass. There are multiple metastatic deposits in the liver (white arrows) and lungs (arrowheads)

The imaging characteristics of renal angiosarcoma can be difficult to distinguish from renal cell carcinoma. The commonest pattern is a large mass (over 5 cm) arising from the kidney associated with peripheral rim enhancement with a non-enhancing centre suggestive of necrosis [65]. MRI may show a striated appearance of the tumour with alternating low and high T2 signal, correlating with haemorrhagic necrosis and solid tumour seen on histology (Fig. 10) [65].

Other uncommon sites of angiosarcoma reported in the literature include the gastrointestinal tract, urinary bladder, testes and penis [46].

### Metastatic patterns of angiosarcoma

Angiosarcomas spread haematogenously, most frequently to the lungs, followed by liver and bones, but spread to the brain, lymph nodes and spleen have also been reported [3, 5–7].

**Fig. 11:**
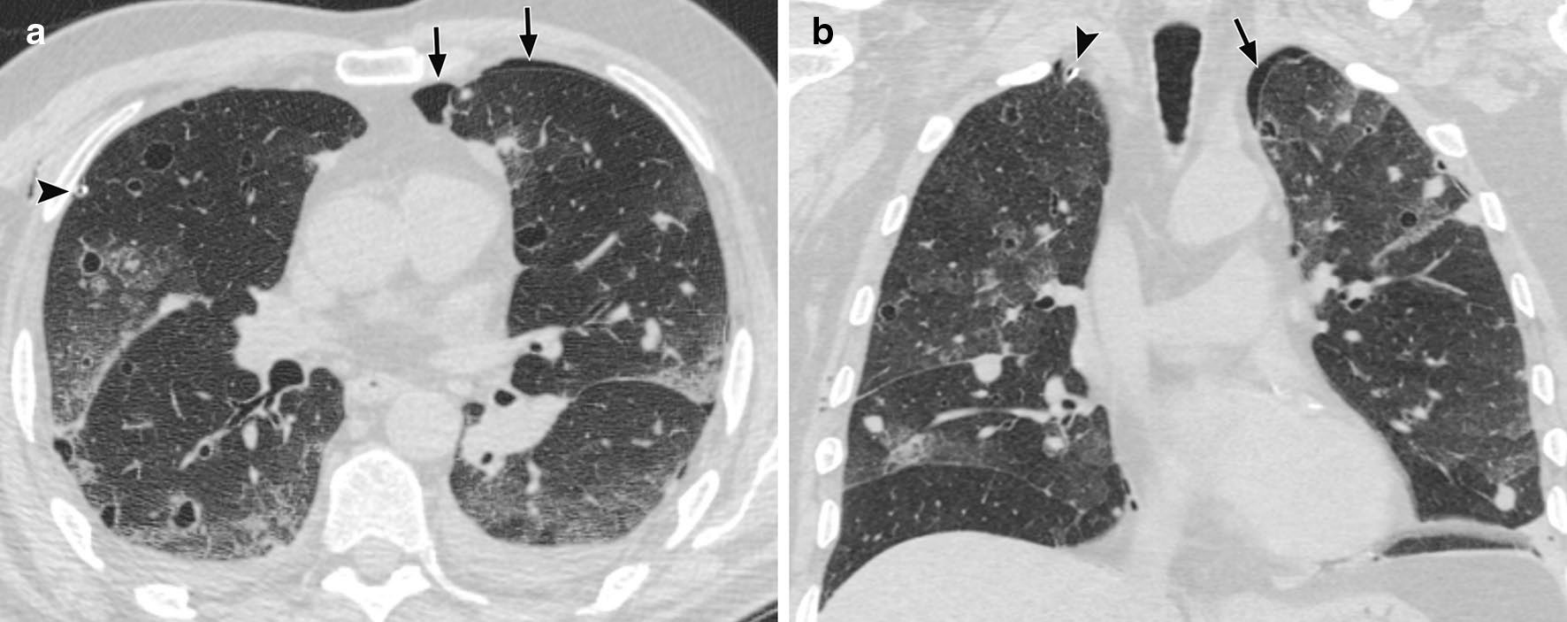
65-year-old with radiation-associated angiosarcoma of the parotid gland and pulmonary metastases. **a** Axial and (**b**) coronal CT (lung windows) images show multiple nodule and cysts in the lungs, some of which are surrounded with ground-glass changes. There is a small left-sided apical pneumothorax (arrows). There is also a chest drain within the right pleural space due to a previous right pneumothorax (arrowhead)

The most common CT findings for lung metastasis are multiple pulmonary nodules (63–85%), followed by multiple cysts which can either be thick or thin-walled (21–58%) (Fig. 11) [67, 68]. Nodules can also develop into cysts, and there is cystic enlargement during the course of the disease [67, 68]. Some cysts may have air-fluid levels within them due to abrupt enlargement and haemorrhage [67, 68]. There are a number of possible explanations for the development of nodules into cysts. Infiltration of malignant cells into the walls of the bronchioles can result in small airway obstruction and cystic distension from a ‘ball-valve’ mechanism [67, 68]. Cyst formation can also occur due to excavation of a solid nodule through the discharge of necrotic material [67, 68].

Other common CT findings include the presence of ground glass change surrounding the nodules or cysts (CT halo sign) corresponding to alveolar haemorrhage, which were demonstrated in 25–58% of patients in previous studies (Fig. 11) [67, 68]. This finding is also commonly seen in a wide variety of other conditions such as fungal infections and metastases from other hypervascular tumours such as melanoma, choriocarcinoma and osteosarcoma [69]. The development of haemorrhage is most likely related to the fragility of the neovascular tissue which is susceptible to rupture [69]. Other related findings on CT include pneumothorax and/or pleural effusions which are seen in up to 48% and 79% respectively [67, 68], with the former seen far more frequently with cysts [68].

**Fig. 12:**
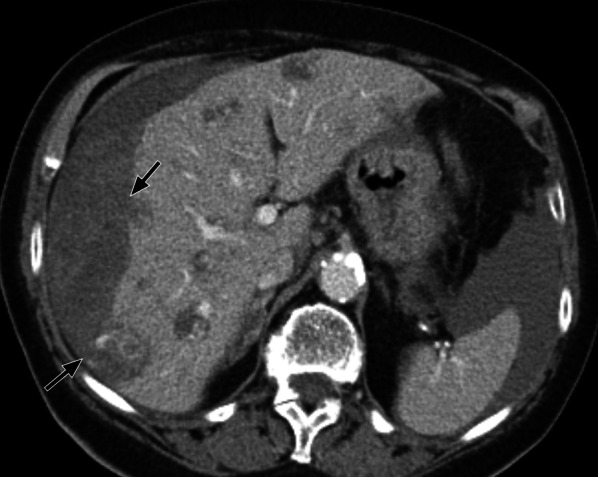
79-year-old woman with radiation-associated angiosarcoma of the breast. Axial contrast-enhanced CT shows multiple liver metastases which are of predominantly low attenuation with central and peripheral areas of enhancement. Haemoperitoneum is seen adjacent to the peripheral metastases (arrows)

CT appearances of liver metastasis in angiosarcoma are typically multiple hypoattenuating lesions with foci of enhancement peripherally or centrally (Fig. 12) [70]. Multiple cystic lesions with fluid–fluid levels can also be seen in 31% of patients, with interval enlargement during the course of the disease [70]. The low attenuation and cystic appearances of liver metastasis correspond with haemorrhagic necrosis on histology. Haemoperitoneum has also been reported in a small number of cases (15%) [70].

There is very little literature available describing the radiological findings of bone metastases from angiosarcoma. In our experience, bone metastases from angiosarcoma can be occult on CT, and MRI is often needed for the evaluation of bone metastases.

## Diagnostic approach

The diagnosis and management of angiosarcoma require a multidisciplinary approach in a specialist soft tissue sarcoma setting given the complexity of the diagnosis and aggressive behaviour of the tumour. The spectrum of imaging and clinical presentation is wide given the heterogeneity of the disease. Imaging alone is not reliable in differentiating soft tissue sarcomas from other benign and malignant lesions [71], although certain clinical features such as the anatomical location and known risk factors (e.g. previous radiotherapy, longstanding lymphoedema) can provide important indicators to narrow the differential diagnosis. A biopsy is recommended to confirm the diagnosis [20], and it is crucial for the biopsy to be evaluated by an experienced specialist sarcoma pathologist [72]. Superficial and cutaneous presentations of the disease can be diagnosed with a punch biopsy or free-hand clinical biopsy. For deeper lesions which are not easily accessible, a percutaneous core biopsy should be performed under imaging guidance to avoid inadvertent damage to adjacent structures. A 14-gauge core needle biopsy is preferred at our institution, with at least four cores taken and in different directions within the tumour to ensure representative tissue is obtained [72]. For hepatic and splenic lesions, the role of percutaneous biopsies is controversial due to the risk of major haemorrhage following the procedure [14, 48, 59], although a number of studies have indicated that the risk of serious complications may be lower than previously described [73, 74]. A biopsy of the liver or spleen should therefore be considered carefully. Imaging may be utilised for biopsy planning, particularly if the tumour is very necrotic and targeting of the remaining viable tissue is crucial [72]. Imaging with CT or MRI can also be helpful to determine the extent and multifocality of the disease before surgery [1]. CT chest is often undertaken to exclude metastatic disease, which would influence treatment options [20]. In our experience, the imaging features of pulmonary metastases can be very striking and should provide an important diagnostic clue to the radiologist.

## Treatment

Radical surgery is often the primary treatment of choice, ensuring wide margins are achieved, but positive margins are common due to the invasive and multifocal nature of the disease [5–7]. For angiosarcoma of the extremities, isolated limb perfusion can be a useful technique for carefully selected cases to reduce the tumour size prior to surgery, but this treatment option is only limited to very few sarcoma referral centres [20]. Adjuvant radiotherapy involving large doses (> 50 Gy) and wide field is recommended due to the high risk of local recurrence, although further radiotherapy is frequently avoided for RAS [1]. The role of adjuvant chemotherapy has not yet been proven even though angiosarcomas are moderately chemosensitive and have a high risk of metastasis [1, 20].

Cytotoxic chemotherapy is the primary treatment option for metastatic angiosarcoma with a number of agents used including taxanes, anthracyclines, gemcitabine and ifosfamide [1]. Participation in clinical trials of novel agents should be encouraged. Recent studies have shown the efficacy of checkpoint inhibitors in cutaneous angiosarcoma [75, 76], and a number of prospective trials exploring this approach are in development. The randomised Phase 3 trial of pazopanib with or without an anti-endoglin antibody in advanced angiosarcoma did not show a difference in outcome between the two arms [77]. However, a number of patients derived durable clinical benefit in both arms, and it is clear that there is a need for functional imaging biomarkers in this heterogeneous sarcoma subtype. The use of chemotherapy may, however, be limited given that many angiosarcoma patients are elderly with co-morbidities and at risk of developing toxicity related to therapy. Notably, oraxol, a combination of oral paclitaxel and a P-glycoprotein inhibitor, has shown activity and favourable tolerability in elderly cutaneous angiosarcoma patients [78]. These trials show that it is possible to perform angiosarcoma specific trials with international collaboration.

## Conclusion

Angiosarcomas are rare soft tissue sarcomas associated with a poor prognosis which can affect any age and any part of the body. The most common presentation is purplish cutaneous masses or nodules involving the scalp and face in elderly men. Imaging features are variable and non-specific reflecting the heterogeneous behaviour of the disease, and patients often present with advanced or metastatic disease. Angiosarcoma should be considered in the differential diagnosis if imaging shows metastatic disease in the lungs with nodules and cysts surrounded with ground-glass opacification and hydropneumothorax. Given the rarity and often dismal outlook for these complex tumours, radiology has a crucial role in diagnosis, intervention and staging. A background knowledge of this interesting and challenging disease with its diverse manifestations is essential for diagnosticians in the specialist oncology setting. Correlation with the clinical history, particularly of any specific risk factors such as previous radiotherapy, chronic lymphoedema or specific familial syndromes, is also vital to narrow the differential diagnosis, but the final diagnosis should always be confirmed with histopathology and immunohistochemistry. The management of this complex disease would require a multidisciplinary approach at a specialist sarcoma unit.

## Data Availability

Not applicable.

## Abbreviations

ADC: Apparent diffusion coefficient
AIDS: Acquired immunodeficiency syndrome
CD31: Cluster differentiation 31
CT: Computed tomography
DCE: Dynamic contrast-enhanced
DWI: Diffusion-weighted imaging
EHE: Epithelioid haemangioendotheliomas
ERG: Erythroblast transformation specific related gene
MRI: Magnetic resonance imaging
PET-CT: Positron emission tomography-computed tomography
RAS: Radiation-associated angiosarcoma
UV: Ultraviolet
